# Cultural competence in mental health care: a review of model evaluations

**DOI:** 10.1186/1472-6963-7-15

**Published:** 2007-01-31

**Authors:** Kamaldeep Bhui, Nasir Warfa, Patricia Edonya, Kwame McKenzie, Dinesh Bhugra

**Affiliations:** 1 Centre for Psychiatry, Barts and The London, Queen Mary's School of Medicine and Dentistry, Old Anatomy Building, Charterhouse Square, London EC1M 6BQ, UK; 2 Department of Mental Health Sciences, Royal Free & University College School of Medicine, University of London, UK; 3 Department of Cultural Psychiatry, Institute of Psychiatry, King's College, University of London, UK

## Abstract

**Background:**

Cultural competency is now a core requirement for mental health professionals working with culturally diverse patient groups. Cultural competency training may improve the quality of mental health care for ethnic groups.

**Methods:**

A systematic review that included evaluated models of professional education or service delivery.

**Results:**

Of 109 potential papers, only 9 included an evaluation of the model to improve the cultural competency practice and service delivery. All 9 studies were located in North America. Cultural competency included modification of clinical practice and organizational performance. Few studies published their teaching and learning methods. Only three studies used quantitative outcomes. One of these showed a change in attitudes and skills of staff following training. The cultural consultation model showed evidence of significant satisfaction by clinicians using the service. No studies investigated service user experiences and outcomes.

**Conclusion:**

There is limited evidence on the effectiveness of cultural competency training and service delivery. Further work is required to evaluate improvement in service users' experiences and outcomes.

## Background

Health professionals are now more aware of the challenges they face when providing health care to a culturally and racially diverse population [1]. Despite concern about ethnic disparities of access to culturally appropriate mental health care, and calls for cultural competency training to be mandatory, there is little information about the effectiveness of cultural competency training in mental health settings [2-4]. It is well established that in order to provide culturally competent care, knowledge of cultural beliefs, values and practices is necessary otherwise health practitioners can easily fall prey to errors of diagnosis, inappropriate management and poor compliance [5]. Training curricula for medical, nursing and social work students now generally include lectures and course work on cultural competency in health care provision. Post-graduate training is also being revised (for example in the UK the Royal College of Psychiatrists) to incorporate cultural influences on mental health care. Despite this progress, a recent tragedy in the UK expedited the acceptance of policies to promote cultural competency training. A psychiatric inpatient was medicated under compulsory legislation and died while being restrained following a period during which he was subjected to racial abuse from another patient. The subsequent inquiry concluded that better training was necessary for the management of imminent violence and for staff to develop cultural competence in care provision [3].

Although such recommendations are laudable, there appear to be several problems with such an approach. There is considerable confusion about what constitutes cultural competence. For example, it may be narrowly interpreted to mean better knowledge of the cultural beliefs and practices of a specific cultural group, with little attention to how culture modifies illness perceptions, illness behaviour, and acceptability of specific interventions. Cultural competency is somehow expected to emerge if the racial and ethnic mix of the workforce is representative of the local population. Not surprisingly, working practices following standardised professional trainings remain similar among staff from different ethnic groups because of the common pattern of training. Indeed, a patient and a health professional, ostensibly belonging to the same ethnic group because of shared country of origin, may actually differ in terms of social class, religious practices, languages, and cultural beliefs about illness and recovery. Despite a growing body of health and educational policies that prioritise cultural competency in health care provision, there is surprisingly little agreement on the meaning of cultural competence training or knowledge about its effectiveness.

### Aims

In this review we seek to: define the meaning of cultural competence in mental health settings, describe models of cultural competence which have been evaluated in mental health settings, and assess the evidence for effectiveness by reviewing studies that implemented a model of cultural competence and then evaluated its effectiveness.

## Methods

All accounts of cultural competency published in English since 1985 were identified. This date was applied to ensure relevance to recent practice and profiles of ethnic groups for whom the training is intended to improve outcomes. The searches were undertaken between January 2004 and June 2004. The titles and abstracts of papers were reviewed against inclusion criteria:

• showed implementation of a cultural competence model of mental health care AND

• provided some evaluation data for a cultural competency model of service provision or training AND/OR included an evaluation of adherence to a pre-defined model of cultural competence in mental health services

Papers meeting these criteria were called A papers (listed in Table 1). Other relevant papers were not extracted but read for background information, and for placing some of the findings in a wider context. We included all papers published in English language that were about adults with mental illness. The literature search including the following databases: Ingenta, Medline via Ovid, Medline via Pubmed, Medline Plus, Health Outcomes, HealthPromis, HSTAT, DocDat, National Research Register, NLM Gateway, Cam, ReFer and Zetoc. Research Phrases/terms included combinations of the following: Cultural Competence, Cultural Capability, Cultural Sensitivity, Mental Health, Mental Healthcare, Mental Health settings, Best practice, Cross Cultural Mental Health and Cross Cultural Psychiatry. Websites known to include cultural competency or educational materials were also searched [6-15].

**Table 1:** Descriptive information on study populations, definitions, models of cultural competence, and outcomes

**Study**	**Nature of Evidence**	**Definitions of CC**	**Reference population**	**Models of cultural competence**
Ferguson (2003)	Setting of standards for curriculum for cultural diversity in the years 1999 and 2000. Evaluation of programme of teaching, and of cultural competence: change in attitudes and behaviour	Cultural competence is a dynamic continuum consisting of seven stages: Listen, Elicit, Assess, Recommend, Negotiate (LEARN)	15 New England & New York Medical Schools, USA 137 participants (83 women, 42% family medicine specialists, 52% less than 10 years in practice.	Community Curriculum Model Module 1: CC and the role of the physician Module 2: teaching skills of CC Module3: Moving beyond cultural awareness
Hadwiger (1999)	Cultural competence in critical care nursing practice. Narrative responses to case scenarios used to develop skills, self reflection, and improved quality of care plans	Process of working with patients from different cultural background than one's own To reflect on beliefs and assumptions Negotiate a plan of care without use of stereotypes Problem solving and writing competencies	Nursing Students in a Midwest Community, USA	Campinha-Bacote Model [30][37] 1. Cultural awareness 2. Cultural Knowledge 3. Cultural Skill 4. Cultural Encounters One case over 6–8 week period: 4 case scenarios
Siegel et al (2003) †	Performance measures of cultural competency were selected and benchmarked in 21 health care organisations inUS Delphi exercise: experts asked to rate importance, feasibility and reliability of indicators; these were then reviewed to ensure they addressed CLAS (cultural and linguistically appropriate services) standards set by US Dept. Health and Human Services	The set of congruent behaviors, attitudes, skills, policies and procedures that enable the organization's caregivers to work effectively and efficiently in cross/multicultural situations	Mental Health Care Organizations in the USA Expert panel of four major ethnic groups in US: African American, Hispanic, Asian Indian, and Asian American Survey data from 21 mental health organizations Telephone interviews of services already implementing cultural competency benchmarks. Key informant interviews with 21 best practice organisations: 15 administrative sites and 8 service entities	Phase 1: **develop a framework of key domains **and select performance measures of cultural competence: (1) needs assessment; (2) information exchange; (3) services; (4) human resources; (5) policies and plan, (6) linked to outcomes. **Three organisational levels**: (1) *administrative (*state mental health authority or a managed care entity); (2) *service delivery entity *and (3) *individuals *involved directly or indirectly with the delivery of care. Phase 2: Measures reduced to a manageable size
Kim-Godwin (2001)	Concept analysis by 13 community nurses and nurse experts	Key domains of cultural competence: 1)Caring 2)Cultural sensitivity, 3)Cultural knowledge 4) cultural skills are'	In 1996 scale tested on 192 senior undergraduate and graduate nursing students in two South Eastern US centres. Community Health Nurses in Idaho. In 1998, in depth interviews with 13 nurses (8 community health nurses and 5 community nurse experts).	Culturally Competence Community Care
Kondrat et al (1999)	Semi-structured interviews with 64 workers at 4 different mental health agencies/case record analysis of 24 consumers Sites matched on per capita expenditure, proportion of African American Clients, proportion of minority staff Sites picked where minorities doing better on community tenure (benchmark agencies) and compared with sites where they were not doing so well	The best practice approach is pragmatic, practice driven, and results oriented.	Community Mental Health Agencies in Ohio, USA **Field observations**: Intensive observations of 3 days per week for a period of 2 weeks Then 2 days per week for a period of 2 weeks Then 1 day per week, for 12 weeks **Semi-structured interviews**: administrators (3 or more), team leaders (2 or more), managers, 6 clients, 6 carers at each of the four sites (snowballing technique) **Document analysis: **Case records of 24 consumers (12 African American and 12 Caucasian) for decision nodes in care. " years of entries per client.	Benchmarking Research Model
Kirmayer et al (2003)	Participant observation & analysis of case reports of first 100 referrals	Cultural consultation models suggest a mechanism to address the impact of cultural diversity on mental health problems.	Mental health Service providers, Montreal, Canada	Cultural Consultation Model based on DSM-IV cultural formulation, using cultural consultants and culture brokers. Three options: 1) 1–3 meetings with patient, and brief report, phone calls, case conference to transmit immediate recommendations, subsequent more detailed report; 2) Cultural consultant discusses case with referring consultant without seeing patient directly. Clinical case conference may ensue; 3) Consultant meets with referring community organisations, without directly seeing community members. In a clinical case conference, community organisations express problems in engaging or providing a service for a specific cultural group.
Frusti et al (2003)	Qualitative data: individual (n = 43) senior staff including directors and managers; and focus group interviews with staff, with efforts to include minority groups Quantitative data from documents from the nursing organisation and organisation as a whole, seeking evidence of diversity competence	Diversity Competence Model assessment: diversity competence is defined as an individual's ability to respect each person's uniqueness. Goals of marketplace success, ability to compete, enhanced overall performance, and increased capability of all staff	Nursing Workforce	Diversity Competency Model: 1) Drivers 2) Linkages 3) Cultures, 4) Measurement, 5) all held together by Commitment
Stork et al (2001)	Case study of five US states To assess implementation of cultural competence provisions in behavioural managed care contracts.	Cultural Competence: "Agencies, programs and services that are responsive to the cultural, racial and ethnic differences of the populations they service" (CASSP, 1984) Culturally competent professionals are those who have " the ability to serve individuals of diverse backgrounds" [38]	Exploratory study of how five states of average population distribution and resources implement, monitor and enforce contractual obligations for culturally competent provision in Medical managed care.	State managed behavioural organisations Federal regulations about cultural competence derive from Disability Act of 1990, Civil Rights Act that prohibits discrimination. Interpretation of these rules: translation services, language assistance, quality assurance rules, including grievance procedures, to have capacity and appropriate range of services to serve enrolees, as well as sufficient disciplinary mix, geographic distribution). Extent of contractual provisions, monitoring, flexibility in provider organisation.
US Department of Health and Human Services (HRSA)	Participant observation/group discussions/documentary analysis Developed an assessment profile for organisational cultural competence, and evaluated its performance in health care organisations across a range of size, expenditure, populations served, and cultural competency levels	Cultural competence is a critical factor in providing relevant services to nations growing culturally ethnically diverse population	USA Health Care Organizations	Organizational Cultural Competence Assessment Profile: gives structure, process and outcome indicators for each of the OMH domains of organisational cultural competency: **Model Domains**: 1)Values and attitudes, 2)Cultural sensitivity, 3) Communication, 4) Policies and Procedures, 5) Training and Staff development, 6) Facility characteristics, 7) Intervention and treatment model, 8) Family and community participation, 9) Monitoring, evaluation and research

† Definitions agreed: **Cultural Group**. A subgroup that is from the major racial ethnic groups of African American, Hispanic American, Asian American, American Indian, or from a recent immigration or refugee group. Subgroups can be narrowly defined in terms of worldview, values, rituals, and the like; however, subgroups are most often defined by distinct languages, such as Vietnamese among Asian Americans; or distinct locales of origin, such as Dominicans among Hispanic Americans. Not included are cultural subgroups that have retained their cultural identity in mainstream America, but in ways that do not preclude their participation in US's system of health care and social welfare. Not included were cultural groups defined by physical disabilities, sexual orientation, or other characteristics, as their particular concerns were not in the purview of this project. **Target Population**. The specific part of the general population designated as the population to be served by the administrative or service delivery entity. **Population Area**. The geographical area designated as the area to be served by the administrative or service delivery entity.

Forward and backward citation tracking was undertaken on A papers to identify any further papers of relevance. We also asked two experts to review the search findings, and recommend any other publications. This yielded a PhD thesis and one paper, but neither met our inclusion criteria as they did not include an evaluation. We aimed to include quantitative and qualitative studies. Two researchers reviewed and extracted data from each of the 9 papers; disagreements on the extracted data were resolved by consensus. Information about the studies was extracted and tabulated, including year of study, author, type of study, country of study, and reference populations (Table 1). We undertook a narrative synthesis of the data that is suitable for observational studies where meta-analysis is inappropriate [16,17].

## Results

A total of 1554 publications were identified; of these 109 were selected for further scrutiny on the basis of screening the abstract and titles; only 9 of these met our basic inclusion criteria. These studies implemented models of cultural competence that were evaluated by qualitative or participatory methods, or presented an evaluation of an intervention to improve cultural competency. All studies were based in North America. Many other models of cultural competency were reported in other papers that did not meet our inclusion criteria; we did not review these as there was no evaluation to support them as a model for real services settings. Most of these additional papers expressed opinions or experiences of teaching and training in cultural competence.

### Scope of Papers

Five papers were on cultural competency for physicians and nurses [18-20], multidisciplinary teams [21], and medical students [22]. Five papers included organisational aspects of cultural competency; these referred to the implementation of an assessment and performance framework [4], assessing and implementing measurable benchmarks for performance management [23,24], interpretation of state legislation, contract language and monitoring for impacts on cultural competency [25]; one paper explored organisational drivers that promote change, whilst ensuring measurement of performance, and that there was a change of organisational culture; this paper also explored how organisations integrated different programmes of activity [16]. One government initiative [4] relied on standards set by the Office of Minority Health [26], called the Culturally and Linguistically Appropriate Services Standards (or CLAS Standards; see Table 1).

### Methods Used in Studies

The study methods varied widely, with outcomes that varied across studies; most studies used an action research process, and none used a randomised control trial design. The methodological variability and reliance on exploratory designs precluded meta-analyses, and even quality assignment, as some studies either did not report their analytic methods in enough detail or evolved their methods during the study. Some only measured adherence to a template of cultural competence, rather than the clinical outcome of adherence to a cultural competency model.

### Definitions of Culture Competence

The definitions proposed in each of the 9 papers were tabulated (Table 1). We present here a synthesis of the key characteristics. Cultural competence included a set of skills or processes that enable mental health professionals to provide services that are culturally appropriate for the diverse populations that they serve. This definition was focussed on an outcome, and included attention to obvious language differences in the consultation, as well as how culture influences attitudes, expressions of distress, and help seeking practices. Consequently, it was suggested that clinical procedures and policies should reflect these. Showing respect for patients' cultural beliefs and attitudes was an important component, especially when their views opposed or differed from the professionals' views. Emphasis was given to a genuine willingness and desire to learn about other cultures, rather than this simply being a managerial requirement. The definitions indicate a common aim, to increase performance and the capabilities of staff when providing service to ethnic minorities. Most studies gave a definition of cultural competence before their evaluation, but one study [25] reported that different definitions were used in different US states (see Table 2).

**Table 2:** Main findings: evaluation and outcomes

**Study**	**Evaluation**	**Outcomes**
Ferguson (2003)	Likert ratings (1–5) of overall value, clarity of objectives, instructor effectiveness For second cohort (2000, N = 55):	Showed high scores on all of these: means 4.1 to 4.4 for each domain, and for two year bands 1999 and 2000 : Intention to change: M1: 5.4% (n = 55), M2: 48.1% (n = 54), M3: 30.2% (n = 43) Actual Change in behaviour: M1: 16.6% (n = 42), M2: 21.4% (n = 42), M3: -
Hadwiger (1999)	Cultural Sensitivity (40% of course marks) Evidence of context of own cultural background considered Ethnocentric attitudes Power orientation Egalitarian relationship Trust in relationships Respect for patient during hypothetical negotiations Manner of addressing hypothetical patients Accuracy of content (30% of course marks) Process (30% of course marks)	Nursing students were able to become more aware of how their own culture affects the nursing care Able to refine cultural competence skills using hypothetical cases and narrative writing Actual marks or origins of students not given
Siegel et al (2003) †	For each level and domain, experts identified key performance indicators identified, performance measures defined, and data sources outlined. 163 indicators 231 measures Without a formal commitment to the development of a process and the dedication of resources for this effort, cultural competence would be difficult to achieve. Reduced to 85 measures	Administrative: *Services.53% *had put into place services that had been adapted or developed for specific cultural groups. *CC Outcomes. 60% *of administrative entities indicated that outcome measures could be analyzed for specific cultural groups. *CC Training and Education.* *73% *indicated staff members receive ongoing education and training related to CC. 87% selected, developed, and/or provided CC training materials to agencies under their purview but only one provided financial assistance to agencies under its purview for conducting CCT *Services. 87% *of the service entities indicated that they had services adapted or developed for specific cultural groups. 29% of these, providing culture-specific services was the mission of the agency; while for the remaining 71%, culture-specific services had been put in place in response to the perceived needs of clients in the community. *CC Training and Education*. 75% indicated that staff of receive ongoing education and training on CC. 87% said all new employees receive CC education and training as part of their orientation. 75% said that professional education (for example, grand rounds) included racial/ethnic/cultural issues. *CC Outcomes*. Outcome measure data were collected inconsistently at the five agencies responding to this question, but all conducted consumer satisfaction surveys. Sixty percent of those responding indicated that the outcome measures could be analyzed for specific cultural groups. 50% said that CC was included in staff performance evaluations.
Kim-Godwin (2001)	Literature review and concept analysis lead to 3 constructs that were evaluated: 1) health care systems, 2) health outcomes, and 3) cultural competence scale ratings.	In factor analyses, cultural knowledge emerged as a components of cultural sensitivity and cultural skills All 13 participants reported that cultural competent care resulted in positive health outcomes in their practice. Specifically, increases in prenatal visits, higher rates of immunization, reduced morbidity and mortality, increased compliance, increased trust, increased self worth, more interest in promoting health. (Actual accounts not presented, only surmises findings).
Kondrat et al (1999)	Nature of interactions between service providers and Caucasian and African American consumers with SMI Themes: Types and locations of service delivery Structure of delivery services Formal and informal organisational culture Decision making process Perceptions of interactions, processes and decisions Analysis based on 700 observations across four sites Constant comparison analyses	All four agencies incorporated policies to support diversity, yet outcomes for diverse clients varied. **11 clusters of activity**: ***Differentiating: B > C*** 1. Agency work culture: pro-agency culture: 2. Openness/boundary flexibility 3. Prevalent supervisory style: consistent, pro-active, and supportive 4. Team functioning and decisions ***Non-Differentiating*** 5. Attitudes towards clients: 6. Demonstration of programme commitment to diversity 7. Level of acceptance 8. Diversity as a clinical issue 9. Clinical orientation 10. Level of interdisciplinary work 11. Organisation of service There was little evidence that race or culture was routinely considered in making treatment decisions
Kirmayer et al (2003)	Participant observation of the first 100 referred cases. 29 referring clinicians for 47 cases completed service evaluation information	Specialized cultural consultation services can play a major role in educating clinicians and in developing innovative intervention strategies Cases seen by the team demonstrated the impact of cultural misunderstandings: incomplete assessments, incorrect diagnoses, inadequate or inappropriate treatments, and failed treatment alliances. 86% of clinicians referring patients to the service reported high rates of satisfaction, but many indicated a need for longer term follow up. 41%: increased knowledge of social, cultural or religious aspects of cases 21%: increased knowledge of psychiatric or psychological aspects of their cases 48% : improved treatments 31%: improved communications, empathy, understanding, therapeutic alliance 14%: increased confidence in diagnosis, treatment Dissatisfaction with: 14%: lack of treatment or more intensive follow up 14% unavailability or inappropriateness of recommended resources 10% concerns about the cultural appropriateness of the cultural broker 10%: too much focus on social context rather than psychiatric issues For 21 cases, some aspects of the recommendations were not implemented: patient non-compliance (13), lack of staff or resources (9), spontaneous improvement (7).
Frusti et al (2003)	Consultant employed to assess drivers, linkages, culture and measurement strengths and weaknesses of organisation	Drivers: 1) nursing diversity committee promotes supportive work environment by sponsoring educational activities & newsletter 2) Nursing recruitment and retention committee 3) Transcultural patient care committee, provides up to date resources about influence of culture on health Linkages:1) Managers and staff share department committee responsibilities, and feed into a shared decision making process 2) Nursing and human resources departments conduct annual planning to identify shared goals, and recruitment targets national and local nursing organisations 3) Summer intern programme to recruit under represented groups Culture: 1) education and orientation to culture of nursing department, leadership roles developed; focus groups indicated managers are trusted, 75% of participants said they were set up to succeed by their nurse mangers 2) primary values: needs of patients first, best nursing care in the world Measurement: Recruitment data, retention data, compared with national benchmarks
Stork et al	Used data from Rosenbaum (1999) study of cultural competence in manage care contracts. Analysis of contract excerpts for cultural competence definitions, and requirements for service provision. Open ended interviews with officials in five states to examine written cultural competence requirements. Purposive sample of states that 1) that had contract with cultural competence provisions 2) more comprehensive requirements than other states, reflecting early implementation 3) were average in resources and populations 4) had officials who could talk in depth about contracts Rosenbaum reported on 37 states, of these 27 had cultural competency requirements, and 10 met criteria. : contract language comprehensive, 2) specific wording about practices rights to culturally competent services States selected because of geographic, ethnic and racial diversity Interview: definition of CC Contract language/standards Methods to measure and enforce standards Methods to track cultural competence Methods to track consumer enrolment/satisfaction/service use by ethnic/racial groups	Lack of indicators for cultural competence, reluctant to enforce existing standards, disagreement over costs, lack of constituency in training and tracking 4 of five states included their own definitions of cultural competence in their contracts • Relate to client with sensitivity, understanding, respect for clients' culture • Understanding social, linguistic, ethnic and behavioural characteristics of a community or a population and the ability to translate systematically, that knowledge into practices in the delivery of services-identify and value difference, acknowledge interactive dynamics of cultural differences, continuously expand cultural knowledge/resources, collaborate with community re provisions and delivery, commit to cross cultural training, develop policies to provide relevant, effective, programs for diverse populations • Ability to serve individuals of all ages, ethnic groups, in a manner appropriate to their age and unique cultural background. • A set of congruent behaviours, attitudes and practices and policies that are formed within an agency and among professionals that enable the system, agency and professionals to work respectfully, effectively, responsibly, in diverse situations. Essential elements include: valuing diversity, understanding dynamics of difference, institutionalising cultural knowledge, and adapting to and encouraging organisational diversity. Themes identified: contract language, contract deliverables, procedures for monitoring and oversight, data collection, provider assessments Contractual deliverables: submit a plan to include translations of written material and access to interpreters at no extra cost, legally mandated. Oversight/agency: assign responsibility to a specific agency. Oversight mechanisms: readiness reviews, site reviews, before roll out. Complaint tracking, consumer satisfaction surveys. Collecting client data: three stated did not collect enrolment data, disenrollment, provider changes, service use or satisfaction by race/ethnicity. Two states can assess requests to change provider by ethnicity, and whether change requested is a result of language problems. None of the states used the cultural data on their client to indicate lack of cultural competence. No state asks clients to rate their cultural competence of provision Penalties: None, and none are enforced. Assessment of CC: determined by provider to MCO/MBHO documentation of training, available ;personnel, representative services as contract deliverables dictate.
US Department of Health and Human Services (HRSA)	Organisational cultural competence assessment profile assesses domains, focus areas and indicators Domains: As in Table 1. For each domain there are Indicators which have a) structure, b) process and c) outcome	Findings suggest that the Assessment Profile can be useful even in its current form as an organizational framework and a guide to an organization's own development of indicators and measures of cultural competence CC must be integrated into other organisational domains of activity Organisational values must be tackled first. Structures, process and outcomes agreed for each of the subheading: **Organisational Values**: Leadership, investment and documentation, Information and data retrieval for cultural competence, Organisational flexibility, Community Involvement and Accountability, Board Development, Policies **Planning, monitoring, evaluation**: Client, community and staff inputs, Plans and Implementation, Collection and use of cultural competence data **Communication**: Understanding communication needs of clients, Culturally competent oral communication/written/other communication, Communication with community, Organisational communication **Staff development**: Training commitment, Training content, Staff Performance **Organisational infrastructure**: Financial, Staffing, Technology, Physical facility characteristics, Linkages **Services/Interventions**: Client family community input, Screening/assessment/care planning, Treatment and follow up

### Mandatory or Discretionary

Table 1 &2 set out the key components of the models and present the outcome data. The studies of individual professions took an educational approach, subjecting each group to an analysis of how best to teach and learn about culture: the key findings include the need for a desire to learn about other cultures and that this could not be mandated. Three papers recommended that training be discretionary [22,18,24], whereas, like UK policy, one paper recommended a compulsory process [23]. Actual encounters with other cultural groups were considered important in all studies.

### Teaching and Learning Methods

Only three studies published their teaching and learning methods. One model of cultural competency recommended participant observation, analysis of case reports, consultation and conferences around specific clinical problems [19]. Another [18] recommended discussing and writing about case histories and paying attention to the narratives. Hadwiger's model was developed for nursing working in critical care settings; this deployed interactive lectures and small group teaching with role-play exercises and patient centred interviews to enhance cultural understanding [20]. Only three studies actually followed up subjects to assess changes in behaviour or adherence to a model of cultural competency following an intervention [19,22,23].

### Organisational Processes

Four studies evaluated organisational approaches [4,23-25], but each study focussed on different processes. Siegel et al developed performance indicators and tested them for feasibility and value within a performance framework for 21 health care organisations [23]. Kondrat et al identified characteristics of better performing culturally competent organisations (called benchmark agencies), where these distinguished them from less culturally competent organisations (comparison agencies): a pro-agency attitude among staff, openness and flexibility of provision, consistent, pro-active and supportive supervision, and team based functioning and decision making were all essential [24]. This study also showed that race and culture were rarely considered in care provision.

The US Dept of Health and Human Services developed a performance framework using the nine domains for cultural competent health care provision proposed by the Office of Minority Health [26]. These include organisational and individual level processes, including a performance framework for culturally competent commissioning and to assess the service impacts (see Table 1).

One US study evaluated how legal requirements in five US states for cultural competence in provider organisations are reflected in contract language, monitoring for adherence to the principles of cultural competency, and in the efforts to enforce adherence [25]. Although four states did include language support, for example, interpretation services, staff capacity and training, none of these contractual expectations were enforced, and there were no penalties for non-adherence.

### Quantitative Outcomes

Only three studies gave quantitative outcomes [21-23]. These showed changes in 'intention to modify practice' following training (30%) and actual changes in behaviour (20%) following training [22]. There was significant (86% of practitioners) satisfaction with the consultation model [21]; 48% reported better treatment, and 31% expressed improved communication, empathy, understanding and therapeutic alliance. There were concerns that not all the recommendations could be followed due to limited resources. A lack of resources and recommendations that were unrealistic were sources of dissatisfaction among clinicians. Siegel et al reported high levels of training and education in administrative and service delivery aspects of service provision (73–87%) as well as a commitment to culturally appropriate services [23]; 87% of the services were adapted or developed for specific cultural groups; 29% of these, provided culture-specific services; for 71% of these culture-specific services had been put in place in response to the perceived needs of clients in the community.

## Discussion

The limited evidence recommends: a) specific processes and forms of learning for practitioners, b) in the context of a culturally competent provider that is c) commissioned and performance managed according to agreed benchmarks. The studies were based in the US or Canada, raising questions about the transfer of knowledge between these and other countries. For example, the managed care and insurance based service models in the US may not translate well to contexts where the services rely on public funding.

The histories of migration to each country will also differ; the emphases given in each country to specific forms of citizenship may favour the adoption of special services or propose that immigrants should assimilate and adapt themselves [27]. Furthermore, histories of colonial rule and positive expectations of each country's response to immigrants from the colonies may lead to disappointment and thwarted aspirations, alongside discrimination that all culminate in particular forms of discourse on cultural competency. For example, in the UK there has been an emphasis on discrimination and racism [27,28]. However, there are general lessons for work in a multi-cultural society and these will now be discussed.

### Individual level cultural competence

The findings suggest that a culturally competent person is able to acknowledge, accept, and value the cultural differences of others. That is, such a person has the knowledge and skill that enable him or her to appreciate value and celebrate similarities and differences within, between, and among culturally diverse groups [29,30]. The 'LEARN' model emphasised more specific skills: Listen, Elicit, Assess, Recommend and Negotiate [22]. The voluntary desire to become culturally competent was seen to reflect an important general attitude towards work with culturally diverse groups [30].

Several sequential stages were identifiable in the pathway towards cultural competence. A developmental process was proposed moving from cultural awareness to improved cultural knowledge and improved skills through encounters [19,20]. This developmental process involved practitioners looking within themselves to reveal expectations about whether others should adapt to our institutional norms and culture [16]. This reflexivity is necessary to develop empathy through a better understanding of the patient's predicament [31], avoid assumptions and stereotypes [21], and to be aware of ones own attitudes and prejudices [32]. Empathy relies on precise communication of emotional experiences and worries, despite language barriers or communication through an interpreter. Indeed, with the right attitude to develop skills, and the aptitude to contain uncertainty, contradictions in communications can be positively harnessed to improve the outcome of therapies [33].

#### Teaching & Learning Methods

The importance of training and education was highlighted. However, there was little information about appropriate content or learning methods in order to optimise learning and teaching impacts on practitioners' knowledge and skills, nor was there information on whether medical or other mental health practitioners require distinct approaches. Few publications evaluate teaching methods and the content of programmes for medical students and other health professionals. This is quite surprising considering there is acknowledgement of the need to examine policies and procedures regarding cultural sensitivity and competence to improve the experiences of black and ethnic minority services users [34].

Reviewing the literature reveals that there were no instances of enforced changes within mental health services. Materials to teach cultural competence maybe limited, but there are recommendations and materials available both in the US and UK to develop programmes [34]. Regrettably, as our review shows, few of these have been subjected to any stringent evaluation of outcomes. Different methods for teaching cultural competence include:

• Lectures: these convey lots of information and are cost effective.

• Case study discussion: these elicit many views, and participant interactions occur and challenge behaviours and attitudes.

• Role-play reveals hidden attitudes and challenges behaviours.

• Video materials and video feedback: this enables portrayal of many perspectives, demonstrates non-verbal communication, and raises awareness.

#### Curriculum Content

Welch divided training content into three areas, knowledge, awareness, and skills [35]. Knowledge focuses on the perspectives of illness and healing, learning about different views of illness and healing. Concepts and definitions of race, culture ethnicity, and the role of power are important to define. This also covers seeking to understand the family and community structures and functions. Awareness of difference and an ability to discern different health and illness beliefs were essential alongside challenging stereotypes and assumptions. Skills that were recommended focussed on social and language barriers in healthcare. An alternative approach is to use of film as a resource for cultural competency training. Like the studies that used case reports, consultation, and thoughtful discussion, the use of film and the arts can help explore the limitations of existing theories about race and ethnicity [36]. This approach brings to the fore the individuals' stereotypes that may shape assessment and clinical management recommendations. Policy and organisational constraints on individual practice can also then be discussed if they are witnessed to obstruct innovation.

### Organisational Cultural competence

The literature revealed several domains of organisational cultural competency including attention to organisational values, training and communication. Cultural competence at the organisational level must be embedded in the infrastructure and ethos of any service provider. Culturally competent organisations actively design and implement services that are developed according to the needs of their service users. This involves working with others in the community, for example traditional healers, religious and spiritual leaders, families, individuals and community groups. Three studies included domains of assessment and performance management [4,23,24]. Clearly, this locates individual training and education in a more complex system of values, finances, policies and contracts [16,25].

However, in the absence of evidence of effectiveness mandatory training is difficult to justify. Thus existing calls for training appear to rely on clinicians' extensive experience of benefits of training, concerns about the uncertainties involved in the care of culturally diverse groups including fears about accusations of discrimination, and political imperatives supported by anti-discriminatory legislation. Careful reading of established training manuals [37,38] show these to be built on complex notions of race, ethnicity and culture, and the interaction with illness experience and behaviour and contexts. Pioneering work is based on experiences of the actual implementation of programmes in many countries in real clinical and service settings [39-41]. In the absence of randomised trials, or clear specification of complex interventions to improve cultural competency, these forms of evidence should be used with care to establish the foundations for future research, training and service development [42].

## Conclusion

Current mental health policies in culturally and racially diverse societies recommend that mental health professionals be cultural competent. However, the response from each country is in part dependent on the specific histories of immigration, and national attitudes towards migrants, citizenship and how to address racial and cultural integration. Cultural competency of care and services may be proposed in quite diverse ways depending on the local context. This mandates the needs for careful research and quality checks on what is proposed and implemented and applied in different countries [42].

This paper shows that although cultural competency training is important, the form it should take and the organisational performance frameworks to assess impacts are under developed. Most studies were exploratory, and few presented quantitative information. Future work should include randomised trials of complex interventions (teaching and organisational policies), alongside evaluations that include service user based assessment of benefit. In order to establish randomised trials, there needs to be agreement on and the development of appropriate outcome measures for educational and service level interventions. These may be distinct from performance measures at a service level, or commissioning frameworks. Investigations could also explore how 'values' in organisations may shift to produce more conducive environments in which anti-discriminatory practice can become embedded and so allow culturally competent care practices to flourish.

## Competing interests

KB is Director of MSc Transcultural Mental Healthcare; NW is Co-ordinator and PE was an MSc student and formerly the administrator for the course. KM and DB: None. The author(s) declare that they have no other competing interests.

## Authors' contributions

The work was supervised by KB and NW. PE obtained all the papers, which were extracted and checked by NW and KB. KB wrote consecutive versions of the paper receiving comments from co-authors. DB and KM were external experts, provided supervision and expert advice, and commented on consecutive drafts of the paper. All authors have read and approved the final manuscript.

## Pre-publication history

The pre-publication history for this paper can be accessed here:


